# Integrating energy justice and economic realities through insights on energy expenditures, inequality, and renewable energy attitudes

**DOI:** 10.1038/s41598-025-12410-y

**Published:** 2025-07-25

**Authors:** Lina Volodzkiene, Dalia Streimikiene

**Affiliations:** 1 Lithuanian Energy Institute, Breslaujos str. 3, Kaunas, LT-44403 Lithuania; 2 Lithuanian Energy Institute, Breslaujos str. 3, Kaunas, LT-44403 Lithuania

**Keywords:** Energy justice, Energy inequality, Climate-neutral society, Climate-change mitigation, Energy justice, Energy access, Energy economics, Socioeconomic scenarios

## Abstract

Energy justice is a cornerstone of the European Union’s pursuit of climate neutrality by 2050, addressing both environmental and societal challenges. This research employs a representative survey to analyze household expenditures on electricity, natural gas, and heating, evaluating the extent of energy inequality and its implications for affordability and access across income groups. The study also explores public attitudes toward renewable energy, focusing on perceived benefits, barriers, and willingness to adopt these technologies. Although the findings center on Lithuania, they offer valuable insights for other valuable insights other EU countries by highlighting shared challenges and opportunities in addressing energy inequality and promoting renewable energy adoption. The results reveal a generally equitable distribution of energy costs but underscore significant economic constraints, as evidenced by widespread reluctance to pay premiums for renewable energy. The research underscores the importance of targeted policies to enhance the affordability of renewable energy, address systemic inequalities, and foster a fair and inclusive transition to a climate-neutral future. These findings contribute to a deeper understanding of energy inequality and its intersection with climate objectives across diverse European contexts.

## Introduction

### The background

The European Union’s pursuit of climate neutrality by 2050 represents not only an urgent environmental imperative but also a profound societal challenge intricately linked to energy justice. Energy justice, at its core, seeks to ensure the fair distribution of energy costs and benefits, equitable access to energy resources, and the meaningful inclusion of all societal groups in decision-making processes. It emphasizes addressing systemic inequities that disproportionately burden marginalized populations, ensuring that the energy transition promotes fairness and inclusivity alongside environmental sustainability. While reducing emissions is critical, it alone cannot achieve the transformative shift required for a sustainable future. Achieving this ambitious climate objective demands a holistic approach that minimizes inequalities, protects vulnerable groups, and ensures equitable access to energy resources. These elements are not just complementary—they are foundational to fostering societal cohesion, building public trust, and maintaining broad support for the energy transition.

Recent crises, including the global pandemic and surging energy prices due to geopolitical tensions, have exposed deep economic vulnerabilities and inequities across the EU. These events have shown that marginalized groups, including low-income households, bear a disproportionate share of energy burdens. Without targeted efforts to address these disparities, public perception of climate policies risks being tainted by skepticism, reducing societal willingness to participate in or support the transition. Equitable access to energy is not just a matter of justice; it is essential for ensuring that the transition is inclusive and resilient.

Energy justice transcends the technical aspects of decarbonization by recognizing that achieving climate neutrality requires active engagement from all societal groups. Addressing inequalities in energy access and affordability enhances societal capacity to adopt sustainable practices. Conversely, neglecting these disparities risks alienating vulnerable populations, fostering resistance to necessary policies, and undermining the credibility of the EU’s climate agenda. For countries like Lithuania, where many communities already struggle to meet basic energy needs, these challenges are particularly pronounced.

By embedding equity into climate policies, the EU not only mitigates the risk of disenfranchisement among the most vulnerable but also strengthens the societal and economic fabric necessary for a successful transition. A fair and inclusive energy system is key to building resilience against future crises, fostering public trust, and achieving an energy transition that is as socially just as it is environmentally sustainable. This approach ensures that the journey to climate neutrality protects all citizens while creating a legacy of shared commitment to safeguarding the planet for future generations.

In addition to energy justice, this study builds on the growing body of literature addressing energy inequality, which focuses on the uneven distribution of energy expenditures, access, and burdens across different socio-economic groups. Energy inequality manifests when households spend disproportionately on energy relative to their income, lack access to clean or affordable energy sources, or face systemic barriers due to housing conditions or regional disparities. Previous studies have applied metrics such as the energy burden index, decile ratios, and the Gini coefficient to quantify these disparities. However, few empirical analyses examine energy inequality based on actual household energy expenditure data. This study seeks to fill this gap by applying Lorenz curve and Gini-based measures to electricity, gas, and heating costs, offering a nuanced expenditure-based perspective on energy inequality in Lithuania.

### Research questions

This article addresses the following research questions, focusing on the specific aspects investigated through the survey and data analysis: (1) What is the distribution of household expenditure on electricity, natural gas, and heating in Lithuania? (2) How can energy inequality be measured using household energy expenditure data? (3) What are the public’s behaviors and preferences regarding renewable energy adoption in Lithuania? (4) What implications do energy inequality and public behaviors have for designing equitable energy policies?

### Research objectives

The primary objective of the research is to evaluate energy inequality in Lithuania by conducting a representative survey of the Lithuanian population to analyze the distribution of household expenditures on electricity, natural gas, and heating. The study seeks to assess the implications of these expenditures for energy affordability and access, as well as to identify disparities in energy costs and their effects on different demographic and socio-economic groups. In addition to examining expenditure patterns, the research aims to explore public attitudes toward renewable energy. This includes investigating willingness to adopt renewable technologies and identifying barriers to their implementation. These insights focus on uncovering specific factors, such as cost concerns or lack of access, that may hinder the transition to cleaner energy sources. The study also examines how energy inequality interacts with broader climate objectives, such as improving energy efficiency and promoting equitable access to sustainable energy solutions.

Although the reduction of greenhouse gas emissions is part of the overarching context, this research does not directly measure emissions but instead provides insights into how addressing energy inequality can support progress toward these goals. While Lithuania is the focal point, the findings contribute to a broader understanding of energy inequality within the European Union, offering insights that are relevant to similar challenges faced by other member states.

### Research methods

A representative survey of the Lithuanian population was conducted to collect empirical data on the extent and regional distribution of energy inequality. The survey consisted of two parts. The first part focused on household energy expenditures, where respondents provided data on their monthly spending for electricity, natural gas, and heating. This data was analyzed to assess the extent and regional distribution of energy inequality, utilizing tools such as Gini coefficient calculations and Lorenz curve analysis. The second part examined public attitudes and behaviors related to energy efficiency and renewable energy adoption, including willingness to pay for renewable energy, resource conservation practices, and preferences for energy-efficient technologies. Conducted through direct, in-person interviews with 1,000 respondents, the survey employed stratified probability sampling to ensure proportional representation across demographic groups such as age, gender, and geographic location. This comprehensive approach offers valuable insights into the relationship between energy spending patterns, public attitudes, and the broader implications for energy justice and climate policy in Lithuania.

### Research gap and contribution

While numerous studies across Europe have examined energy poverty through income-based indicators, there remains a lack of detailed analysis that focuses on actual household energy expenditures and their distributional implications. In particular, the use of Lorenz curves and Gini coefficients to assess inequality in electricity, gas, and heating costs is underexplored in the context of Lithuania and other Central and Eastern European countries. Additionally, there is limited research that integrates quantitative inequality measures with public perceptions and attitudes toward renewable energy. This study addresses these gaps by applying a dual approach: it combines an expenditure-based analysis of energy inequality with an assessment of public readiness to adopt climate-neutral technologies. In doing so, the research contributes new insights to the fields of energy justice and energy transition policy.

### The practical implication

The research offers significant practical implications for promoting renewable energy adoption and the mitigation of energy inequality. The insights gained can guide the the development of targeted policies and interventions to reduce energy disparities, such as tax credits or grants for renewable energy initiatives. Additionally, these findings can support educational campaigns aimed at increasing public awareness of renewable energy by addressing misconceptions and emphasizing its benefits, thereby encouraging broader adoption. Understanding public attitudes and barriers toward renewable energy also enables the formulation of effective market strategies for renewable energy companies. Furthermore, the research integrates energy inequality with broader climate objectives, including reducing greenhouse gas emissions and achieving climate neutrality, offering actionable strategies for climate action. Although Lithuania serves the primary focus, the results provide valuable insights that are applicable to other EU countries, fostering cross-country learning and collaboration on energy and climate issues. By translating these findings into practical policies and programs, stakeholders can address energy inequality, drive renewable energy adoption, and advance sustainability objectives.

## Theoretical background

The European Union’s ambitious objective of attaining climate neutrality by 2050 is not merely an environmental initiative; it also reflects the importance of energy justice and the degree to which individuals comprehend the significance of climate objectives. The principle of energy justice underpins the EU pursuit of climate neutrality, ensuring that the costs and benefits of the green transition are distributed equitably and that no social group is disproportionately affected by energy policies, costs, or access to the services^1–3^. Achieving climate neutrality requires not only the implementation of just energy policies but also fostering widespread public comprehension of and engagement with these goals. Assessing the public’s comprehension of these objectives and their commitment to climate action is especially important in regions that are experiencing substantial energy inequality because such disparities can hinder the equitable implementation of climate policies and the widespread adoption of sustainable practices^3–5^. Without adequate understanding and engagement, vulnerable populations may lack the resources or motivation to transition to greener energy solutions, exacerbating existing inequalities and undermining the overall effectiveness of the EU’s efforts to reduce greenhouse gas emissions and promote sustainability.

Energy justice is essential for a fair transition to a climate-neutral society, as it aims to address disparities in the accessibility and affordability of energy resources^6–9^. Closely linked to energy justice, energy inequality highlights significant imbalances in the distribution and consumption of energy resources across different demographic groups. the disparities in the accessibility and affordability of energy resources that exist among different demographic groups. These often exacerbate exacerbate social, economic, and environmental disparities^10,11^. Vulnerable populations, including low-income households, rural communities, and marginalized groups, frequently endure a disproportionate share of energy costs while receiving fewer benefits from green policies. Such disparities not only deepen the social divide but also hinder progress toward a sustainable and equitable future^12–15^.

Fostering public comprehension of climate objectives and motivation to contribute to climate initiatives is closely tied to energy democracy and community empowerment, which are integral aspects of energy justice. Public perception plays a crucial role in shaping climate policies, and there is an increasing recognition that the EU’s green transition depends on meaningful public engagement and awareness^16–19^. When individuals feel disconnected from the broader climate agenda, due to a lack of understanding about its significance for the planet’s future or the ways in which individual actions contribute to global efforts, they are less likely to support or participate in these initiatives Although citizens are often aware of climate-related issues, many may not fully grasp the importance of their role in achieving climate neutrality. This observation underscores the need for policies that not only address energy inequality but also enhance community agency and foster a stronger public commitment to climate objectives through inclusive and participatory approaches.

Energy justice is founded on three fundamental dimensions: procedural, recognition, and distributional justice. Distributional justice focuses on the equitable allocation of energy resources, with particular attention on vulnerable populations, to ensure that the green transition does not exacerbate existing disparities^6,20–24^. Procedural justice emphasizes inclusive decision-making, allowing all social groups to actively participate to the development of energy policies that directly affect them^25,26^. Recognition justice ensures that marginalized communities’ voices are not only heard but also respected, addressing their unique needs and vulnerabilities while safeguarding against misrepresentation or inaccurate characterization^21^. This dimension also acknowledges their valuable contributions to the energy transition. Together, these dimensions are crucial for fostering of a sense of collective responsibility for the future and shaping how individuals perceive and engage with climate objectives.

Addressing energy inequality as part of the broader climate agenda is crucial, as evidenced by the gap between the lived experiences of vulnerable populations and the statistics on energy poverty in the EU^27–29^. For instance, the disparity between official data and actual conditions in countries such as Lithuania highlights the extent to which energy poverty remains an underrecognized issue^30^. This gap underscores the need of localized and nuanced energy justice strategies to ensure that all communities can participate in and benefit from the green transition. Tackling these inequalities is not only a matter of equity, but also a practical necessity for achieving the EU’s climate objectives.

Furthermore, energy inequality has extensive environmental consequences^31,32^. While studies on consumption-based carbon emissions reveal that the wealthiest individuals are responsible for a disproportionate share of global emissions, inequality in energy access and consumption also contributes to climate change and environmental degradation. Vulnerable populations often rely on energy sources that are less efficient and more polluting, such as biomass or outdated technologies^12,33^. This reliance perpetuates a vicious cycle in which marginalized communities, despite their relatively low overall emissions, become both contributors to and recipients of environmental damage^34^. Therefore, addressing energy justice is crucial not only mitigating environmental harm and ensuring that future generations inherit a sustainable and habitable planet.

Public participation in energy legislation and climate objectives is an indispensable component of energy justice. The success of climate action will ultimately depends on the public’s understanding of and commitment to these objectives^17,35^. However, public awareness and engagement with climate policies vary widely, with many individuals feeling disconnected from the measures intended to address climate change. This disconnect highlights the need to foster stronger public involvement and understanding to bridge the gap between policy intentions and individual actions. For the EU to achieve its climate-neutrality objectives, a collaborative effort is required involving not only policymakers but also ordinary citizens who recognize the importance of reducing carbon emissions, conserving energy, and investing in renewable energy initiativesy. In democratic societies, the success of climate policies depends on securing widespread public “buy-in” to build the political and societal support necessary for transformative action. Moreover, scholars have linked citizen engagement to the progress and success of energy transitions, as public participation helps to accelerate the adoption of climate policies, reduce resistance, and minimize the risk of protests and pushback. Fostering public commitment to climate objectives requires cultivating a shared sense of responsibility aligning individual actions with collective goals. Citizens must not only informed about the significance of energy justice but also feel empowered to contribute actively to the green transition^6,36^. Ultimately, the EU’s progress toward its climate-neutrality targets will hinge on the full engagement and commitment of its citizens, as public perception of climate goals is a critical factor in success of policies designed to ensure a sustainable and equitable future.

Energy justice is a critical framework for the EU‘s pursuit of climate-neutral objectives by 2050. A successful green transition requires addressing energy inequality, ensuring an equitable distribution of resources, and fostering public commitment to climate goals. Without prioritizing energy justice in policymaking, the path to sustainability risks of neglecting vulnerable populations and exacerbating existing disparities. By incorporating these principles, the EU can build a more sustainable, inclusive, and equitable energy system that benefits both current and future generations.

## Methodology

The methodology is divided into components to address the research questions and achieve the study’s objective. *First*, a representative survey of the Lithuanian population was conducted to collect empirical data on household expenditures for electricity, natural gas, and heating. This aimed to evaluate the relationship between household energy spending and energy affordability, access, and disparities in costs across different population segments. *Second*, the data was analyzed to assess the extent of energy inequality in Lithuania, focusing on variations in household expenditures. *Third*, public attitudes toward renewable energy were investigated, including perceived benefits, barriers, and willingness to adopt these technologies. This comprehensive approach to energy justice considers both the current level of energy inequality and societal attitudes, readiness, and capacity to utilize renewable energy sources. It provides insights into feasibility of achieving climate-neutral goals and highlights the additional efforts needed to safeguard the planet for future generations.

### Survey design and data collection

To determine the degree of energy inequality among households, the research methodology employed a structured survey design that focused on quantifying household expenditures electricity, natural gas, and heating. The survey was conducted by the public opinion and market research center “Vilmorus” at the authors’ request. Data was collected through direct, in-person interviews conducted between November 10 and November 19, 2023, targeting Lithuanian residents aged 18 years and older. A total of 1,000 respondents participated, providing a robust and reliable dataset. The survey utilized probability sampling, specifically stratified sampling, ensuring proportional representation across demographic groups, including age, gender, and place of residence. The sample included 44.2% men and 55.8% women. Respondents ranged across age groups, with 5.6% under 30 years old, 14.8% aged 30–39, 19.3% aged 40–49, 20.3% aged 50–59, 21.4% aged 60–69, and 18.6% aged 70 years or older. Geographically, 19.5% of respondents lived in Vilnius, 10.5% in Kaunas, 5.2% in Klaipėda, and 32.2% in rural areas, with the remainder distributed across smaller cities and towns.

The sample size of 1,000 respondents offered a statistically reliable margin of error of ± 3.1% at a 95% confidence level, based on Lithuania’s population of approximately 2.8 million. However, because not all respondents provided complete data for each expenditure category, the effective sample size for specific analyses varied. For example: (1) electricity expenditures – data was provided by 993 respondents; (2) natural gas expenditures – data was provided by 460 respondents; (3) heating costs – data was provided by 960 respondents. The disparity in response rates reflects differences in energy infrastructure and consumption patterns across Lithuania. Natural gas usage is less prevalent in rural areas and smaller towns, where alternative heating methods, such as biomass or electric heating, are more common. Additionally, urban areas have seen a gradual shift toward renewable energy and all-electric systems, which reduces reliance on natural gas. This divergence highlights the importance of tailoring energy justice policies to address regional and infrastructural differences.

### Data processing and expenditure categorization

Respondents were asked to report their monthly household electricity and natural gas expenses, as well as annual heating costs. Expenditures were categorized, and average spending was estimated by calculating the midpoint of each expenditure range. Total expenditures for each category were calculated by multiplying the number of respondents in each range by the midpoint value. The cumulative percentages were calculated by summing the expenditures sequentially across expenditure ranges to reflect how spending is distributed within the population. Normalized cumulative percentages were then computed by dividing the cumulative totals by the overall total expenditure for each category, resulting in values expressed as percentages between 0 and 100. This normalization provided a clear basis for comparison across different categories and facilitated the creation of Lorenz curves.

### Gini coefficient calculation method

To quantify inequality in household energy expenditures, the study uses the Lorenz curve and the corresponding Gini coefficient. The Lorenz curve plots the cumulative share of total energy expenditure against the cumulative share of households, ranked by their energy expenditure. To compute the Gini coefficient, the following formula is used:

1 $$\:G\hspace{0.17em}=\hspace{0.17em}0.5\:-\:{\int\:}_{0}^{1}y\:\left(x\right)\:dx,$$

2 $${\text{where}}y(x)\,=\,a{x^2}\,+\,bx\,+\,c$$

is the fitted quadratic function approximating the Lorenz curve. The definite integral of this function is calculated over the interval [0,1], and the result is subtracted from 0.5—the area under the line of perfect equality—to obtain the Gini coefficient. The coefficients *a*,* b*,* c* are derived using least squares regression for each energy type (electricity, gas, heating).

### Lorenz curve construction

To visualize the distribution of energy expenditures and assess inequality, Lorenz curves were plotted using these cumulative distribution data. These curves depicted the proportion of total expenditures accounted for by the lowest percentages of the population, providing a clear representation of inequality. The Gini coefficient, a key metric in this analysis, was calculated to quantify inequality. This involved computing the area between the Lorenz curve and the line of perfect equality, with results expressed as a percentage ranging from 0 (perfect equality) to 100 (complete inequality). In all Lorenz curve illustrations, the X-axis represents the cumulative percentage of the surveyed population ordered by expenditure level (from lowest to highest), while the Y-axis shows the cumulative share of total expenditure. This allows us to assess the degree of inequality in how electricity, gas, and heating costs are distributed across households.

This quantitative approach, in conjunction with the examination of public perceptions of renewable energy, provides valuable insights into the obstacles to energy affordability and the preparedness of society to adopt climate-neutral solutions. By combining representative surveys, rigorous statistical tools, and an emphasis on geographic and demographic diversity, the study highlights disparities in household energy expenditures and their implications for energy justice. The findings not only assess Lithuania’s progress toward climate-neutral goals but also offer insights that can be adapted by other EU countries facing similar challenges in achieving energy equity and sustainability.

## Analyzing energy inequality via household expenditure distributions: a comprehensive study of electricity, natural gas, and heating expenses

A representative survey of the Lithuanian population was conducted to collect empirical data on household expenditures related to electricity, natural gas, and heating in order to evaluate the level of energy inequality in Lithuania. This method aids in assessing the relationship between energy costs and household’s ability to afford them, thereby highlighting the financial burden that energy expenses impose on households. By analyzing the data, the study identifies disparities in energy access, consumption, and costs across various demographic and socio-economic segments of the population. The insights gained provide a deeper understanding of the economic challenges households face in meeting their energy needs.

**Household electricity expenses**.

In the questionnaire, respondents were asked what their monthly household electricity expenses are, on average. Out of 1,000 consumers surveyed, 993 provided their responses, while 7 did not specify their spending. The responses of the 993 respondents were distributed as shown in Table 1.

**Table 1:** Distribution of monthly household electricity expenses among respondents.

	Number of respon-dence	Share of respon-dence %	Mid-point (Eur)	Total expenditure (Eur)	% from total	Cumulative population share	Cumulative energy expenditure share
Up to 14 Eur	74	7.5	7	518	1.59	0.0745	0.0159
15–20 Eur	193	19.44	17.5	3377.5	10.36	0.2689	0.1195
21–30 Eur	216	21.75	25.5	5508	16.90	0.4864	0.2885
31–50 Eur	269	27.09	40.5	10894.5	33.43	0.7573	0.6228
51 and more Eur	241	24.27	51	12,291	37.72	1	1

**Fig. 1:**
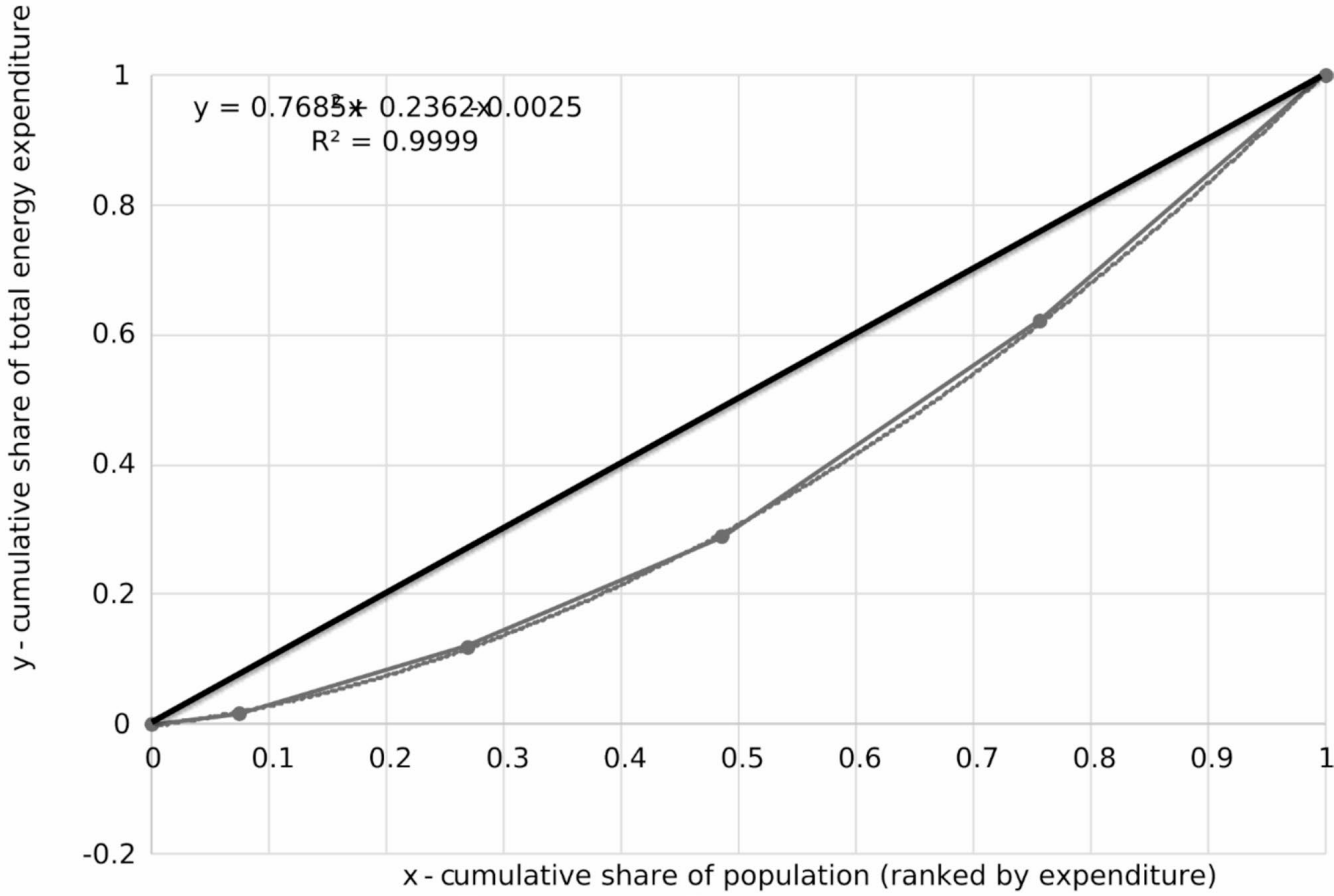
Lorenz curve representing the distribution of household electricity expenditures. The X-axis shows the cumulative share of the population, ranked by electricity expenditure (from lowest to highest), while the Y-axis shows the cumulative share of total electricity spending. The deviation from the 45-degree equality line indicates the degree of inequality in electricity expenses.

Having performed cumulative calculations as indicated above, this enables us to attempt to calculate the Gini coefficient and at least theoretically try to assess energy inequalities on a scale measured by household electricity expenses. The Gini coefficient is a widely used statistical measure of inequality within a population. It is derived from the Lorenz curve, which plots the cumulative distribution of a resource (such as income or expenses) against the cumulative percentage of the population. The Gini coefficient ranges from 0 to 1, where 0 represents perfect equality (everyone has an equal share) and 1 indicates perfect inequality (one person has everything). Higher values signify greater levels of inequality. Thus, the Fig. 1 below graphically represents the level of energy inequality as assessed by cumulative household electricity expenses and their distribution among respondents. In this figure, the x-axis represents the cumulative share of the population, ranging from 0 (lowest-spending households) to 1 (all households), while the y-axis represents the cumulative share of total household electricity expenses, ranging from 0 (no expenses) to 1 (total electricity expenses across all respondents). This visualization allows us to observe the extent to which electricity expenditures are evenly or unevenly distributed among households. A perfectly equal distribution would follow the 45-degree line of equality, while deviations from this line indicate varying degrees of inequality.

To determine the Gini coefficient for electricity expenditures, the definite integral of the fitted Lorenz curve function over the interval [0; 1] must be calculated. The quadratic approximation of the Lorenz curve is given by:

3 $$y\left( x \right)\,=\,0.7685{x^2}\,+\,0.2362x\, - \,0.0025$$

Using the standard formula for the Gini coefficient:

4 $$\:G\hspace{0.17em}=\hspace{0.17em}0.5\:-\:{\int\:}_{0}^{1}y\:\left(x\right)\:dx$$

Each term of the function is integrated separately:

5 $$\:{\int\:}_{0}^{1}y\left(x\right)\:dx=\:{\int\:}_{0}^{1}\left(0.7685{x}^{2}+\:0.2362x\:-\:0.0025\right)dx$$

Calculating each integral separately:

6 $$\:{\int\:}_{0}^{1}0.7685{x}^{2}\:dx=\:0.7685*\:\frac{1}{3}=0.2562$$

7 $$\:{\int\:}_{0}^{1}0.2362x\:dx=\:0.2362*\:\frac{1}{2}=0.1181$$

8 $$\:{\int\:}_{0}^{1}-0.0025\:dx=\:-0.0025$$

Summing the results:

9 $$\:{\int\:}_{0}^{1}y\left(x\right)\:dx=\:0.2562+\:0.1181-\:0.2025=0.3718$$

Therefore, the Gini coefficient is:

10 $$G\,=\,0.5\, - \,0.3718\,=\,0.1282$$

Expressed as a percentage, the Gini coefficient is:

11 $$G\,=\,0.1282 \times 100\,=\,12.82\%$$

This value reflects the degree of inequality in household electricity expenditure distribution among the surveyed population. The obtained results and calculated Gini coefficient of 12.82% for household electricity expenses indicates a relatively low level of inequality in how these expenses are distributed among households. This low value, especially when compared to the country’s income inequality or broader economic benchmarks, suggests that most households have similar electricity expenses, with only minor differences between them. Economically, this could mean that households tend to consume similar amounts of electricity or that their energy needs are fairly uniform. However, this does not eliminate the presence of energy inequality, which can manifest in other forms, such as disparities in access to energy-efficient appliances, heating options, or the ability to invest in renewable energy solutions.

### Natural gas expenditure

In the questionnaire, respondents who use natural gas were asked about their expenditure on it in the last year. Out of 1,000 respondents, 460 provided their expenditures, while 21 did not specify and 519 did not have such costs. The responses of the 460 respondents were distributed as indicated in Table 2.

**Table 2:** Distribution of natural gas expenditures among respondents in the last year.

	Number of respon-dence	Share of respon-dence %	Mid-point (Eur)	Total expenditure (Eur)	% from total	Cumulative population share	Cumulative energy expenditure share
Up to 30 Eur	56	12.17	15	840	35,77	0.1217	0.0358
31–60 Eur	129	28.04	45.5	5869.5	24.99	0.4022	0.2857
61 and more Eur	275	59.78	61	16,775	71.43	1	1

**Fig. 2:**
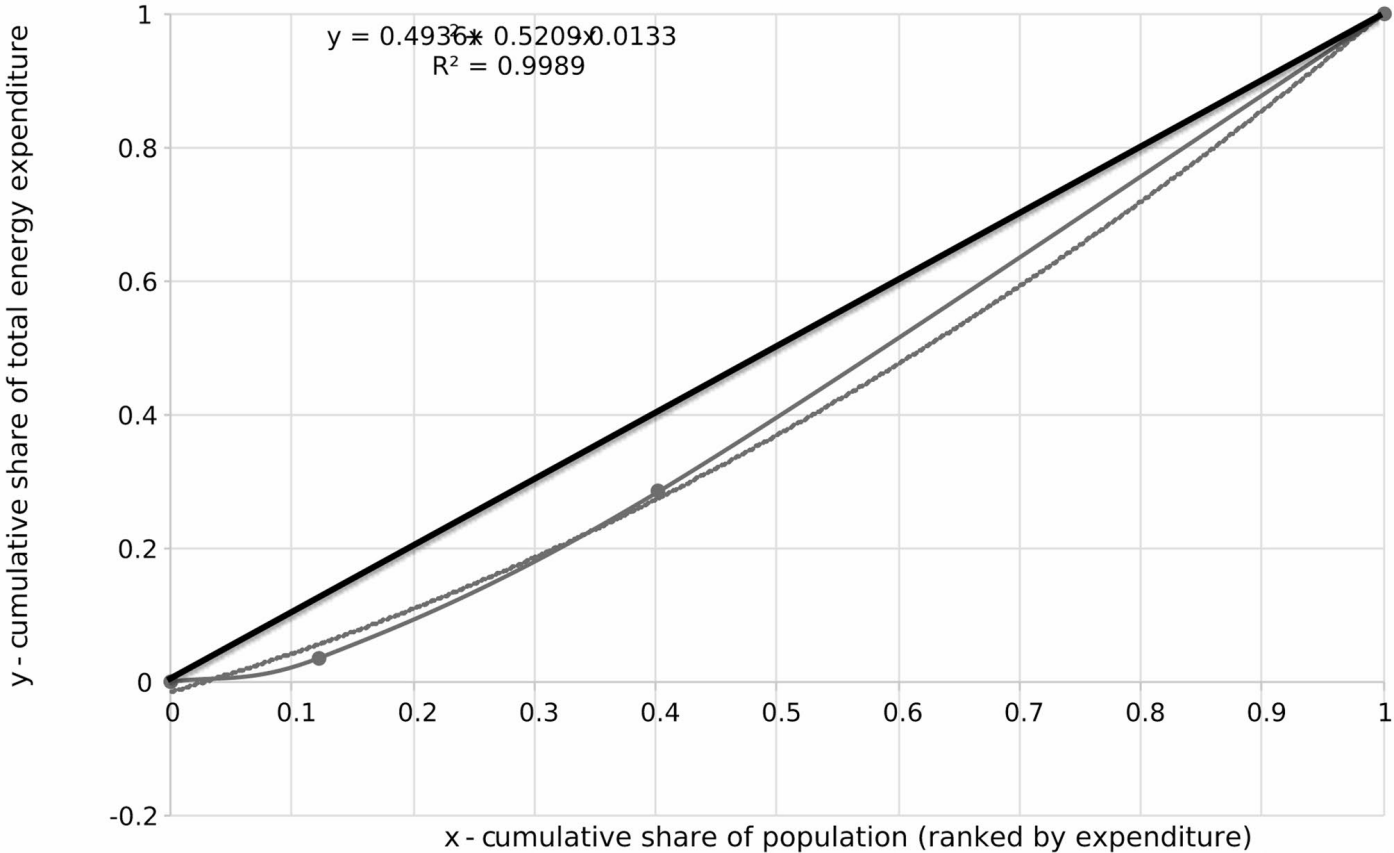
Lorenz curve representing the distribution of household natural gas expenditures. The X-axis shows the cumulative share of the population, ranked by gas expenditure, and the Y-axis shows the cumulative share of total gas spending. The curve illustrates how unequally natural gas costs are distributed across households.

The calculated data enables the plotting of the Lorenz curve (Fig. 2) and the subsequent calculation of the Gini coefficient. The Lorenz curve visually represents the cumulative distribution of natural gas expenditures among respondents. In Fig. 2, the x-axis represents the cumulative share of the population, ranging from 0 (households with the lowest expenditures) to 1 (all households included in the dataset), while the y-axis represents the cumulative share of total natural gas expenditures, ranging from 0 (no expenditures) to 1 (total expenditures across all respondents).

To determine the Gini coefficient for natural gas expenditures, the definite integral of the fitted Lorenz curve function over the interval [0;1] is calculated. The quadratic approximation of the Lorenz curve is:

12 $$y\left( x \right)\,=\,0.4936{x^2}\,+\,0.5209x\, - \,0.0133$$

The Gini coefficient is computed using the formula:

13 $$\:G\hspace{0.17em}=\hspace{0.17em}0.5\:-\:{\int\:}_{0}^{1}y\:\left(x\right)\:dx$$

Each term of the function is integrated separately:

14 $$\:{\int\:}_{0}^{1}y\left(x\right)\:dx=\:{\int\:}_{0}^{1}\left(0.4936{x}^{2}+\:0.5209x\:-\:0.0133\right)dx$$

Calculating each term:

15 $$\:{\int\:}_{0}^{1}0.4936{\text{x}}^{2}\:\text{d}\text{x}=\:0.4936\text{*}\:\frac{1}{3}=0.1645$$

16 $$\:{\int\:}_{0}^{1}0.5209\text{x}\:\text{d}\text{x}=\:0.5209\text{*}\:\frac{1}{2}=0.2605$$

17 $$\:{\int\:}_{0}^{1}-0.0133\:\text{d}\text{x}=\:-0.0133$$

Summing the results:

18 $$\:{\int\:}_{0}^{1}\text{y}\left(\text{x}\right)\:\text{d}\text{x}=\:0.1645+\:0.2605-\:0.0133=0.4117$$

Thus, the Gini coefficient is:

19 $$G\,=\,0.5\, - \,0.4117\,=\,0.0883$$

Expressed as a percentage:

20 $$G\,=\,0.0883 \times 100\,=\,8.83\%$$

This value represents the degree of inequality in natural gas expenditures among the surveyed population. The obtained results and calculated Gini coefficient of 8.835% for the distribution of natural gas expenditures among respondents indicate a relatively low level of inequality. When compared to the country’s income inequality or other economic benchmarks, this value suggests a notably even distribution of natural gas expenditures among the respondents. This means that most households spend similar amounts on natural gas with few extreme differences in spending levels. Economically, this low level of inequality implies that natural gas is relatively affordable and accessible to the majority of the surveyed population. If natural gas were prohibitively expensive or less accessible to lower-income households, a higher Gini coefficient would be expected, reflecting greater inequality. The even distribution of expenditures might also indicate homogeneity in the respondents’ consumption patterns, potentially influenced by factors such as similar household sizes, standardized heating and cooking needs, or uniform pricing and subsidies for natural gas across the surveyed population.

### Heating costs

In the questionnaire, we asked respondents about their heating costs over the last year. Out of 1,000 respondents, 960 provided their costs, while 40 did not specify. The responses of the 960 respondents were distributed as shown in Table 3.

**Table 3:** Distribution of annual heating costs among respondents.

	Number of respon-dence	Share of respon-dence %	Mid-point (Eur)	Total expenditure (Eur)	% from total	Cumulative population share	Cumulative energy expenditure share
Up to 250 Eur	74	20.83	125	25,000	5.06	0.2083	0.0506
251–400 Eur	193	18.02	325.5	56311.5	11.40	0.3885	0.1647
401–800 Eur	216	30.00	600.5	172,944	35.03	0.6885	0.5149
801 and more Eur	269	31.15	801	239,499	48.51	1	1

**Fig. 3:**
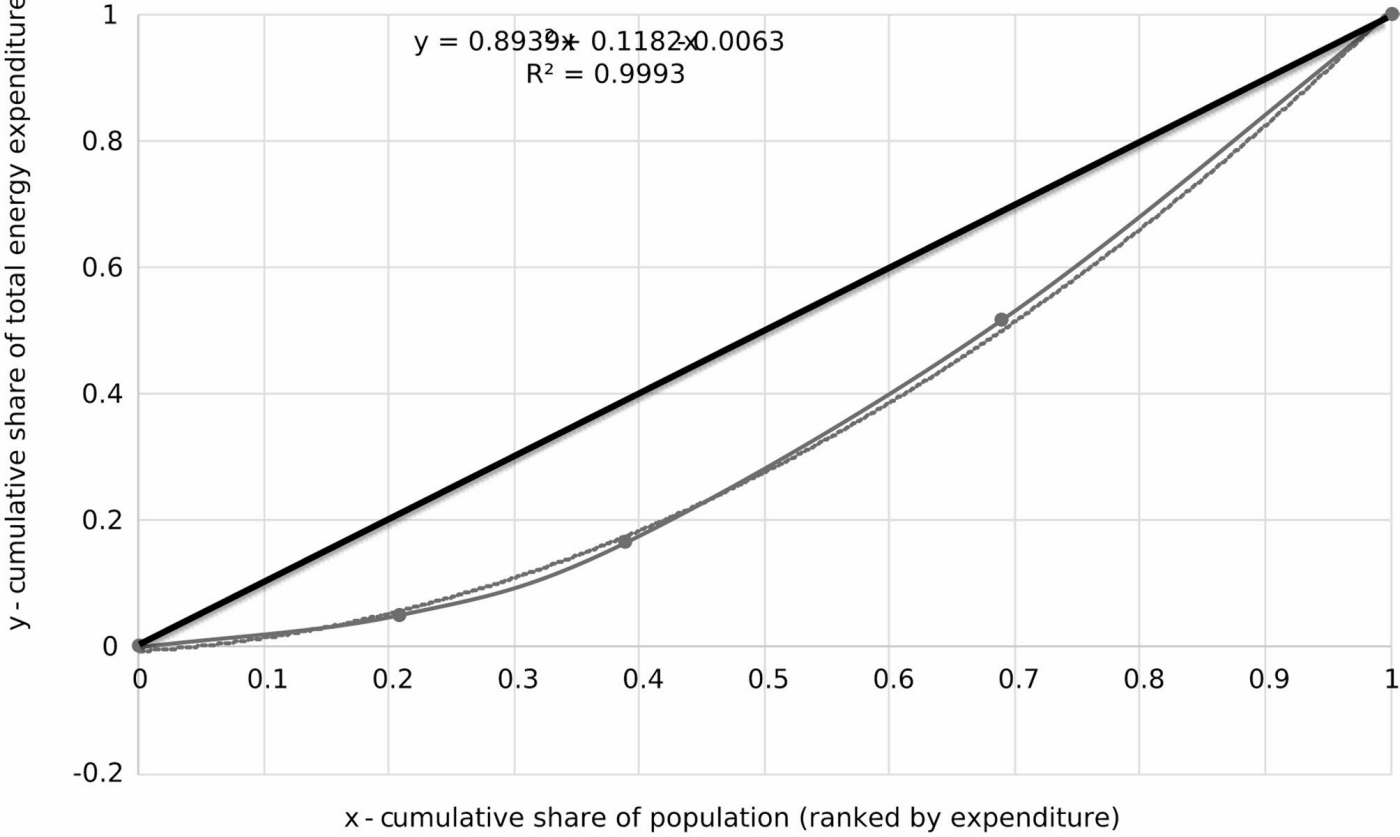
Lorenz curve representing the distribution of annual household heating costs. The X-axis shows the cumulative share of the population, ranked by annual heating expenditure, and the Y-axis shows the cumulative share of total heating costs. The further the curve is from the 45-degree line, the higher the level of inequality in heating expenses.

The calculated data enables the plotting of the Lorenz curve (Fig. 3) and the subsequent calculation of the Gini coefficient. The Lorenz curve visually represents the cumulative distribution of heating expenditures among respondents. In Fig. 3, the x-axis represents the cumulative share of the population, ranging from 0 (households with the lowest heating expenditures) to 1 (all households included in the dataset), while the y-axis represents the cumulative share of total heating expenditures, ranging from 0 (no expenditures) to 1 (total expenditures across all respondents).

To determine the Gini coefficient for heating expenditures, the definite integral of the fitted Lorenz curve function over the interval [0;1] is calculated. The quadratic approximation of the Lorenz curve is:

21 $$y\left( x \right)\,=\,0.8939{x^2}\,+\,0.1182x\, - \,0.0063$$

The Gini coefficient is computed using the formula:

22 $$\:G\hspace{0.17em}=\hspace{0.17em}0.5\:-\:{\int\:}_{0}^{1}y\:\left(x\right)\:dx$$

Each term of the function is integrated separately:

23 $$\:{\int\:}_{0}^{1}y\left(x\right)\:dx=\:{\int\:}_{0}^{1}\left(0.8939{x}^{2}+\:0.1182x\:-\:0.0063\right)dx$$

Calculating each integral separately:

24 $$\:{\int\:}_{0}^{1}0.8939{x}^{2}\:dx=\:0.8939*\:\frac{1}{3}=0.29797$$

25 $$\:{\int\:}_{0}^{1}0.1182x\:dx=\:0.1182*\:\frac{1}{2}=0.0591$$

26 $$\:{\int\:}_{0}^{1}-0.0063\:dx=\:-0.0063$$

Summing the results:

27 $$\:{\int\:}_{0}^{1}y\left(x\right)\:dx=\:0.29797+\:0.0591-\:0.0063=0.35077$$

Therefore, the Gini coefficient is:

28 $$G\,=\,0.5\, - \,0.35077\,=\,0.1492$$

Expressed as a percentage, the Gini coefficient is:

29 $$G\,=\,0.1492 \times 100\,=\,14.92\%$$

This value reflects the degree of inequality in household electricity expenditure distribution among the surveyed population. The obtained results and calculated Gini coefficient of 14.92% for heating costs suggest a relatively low level of inequality in how heating expenses are distributed among the respondents. When compared to the country’s income inequality or other relevant economic benchmarks, this figure indicates a more even distribution of heating costs among households. This means that most respondents likely experience similar heating costs, with only minor differences. A lower Gini coefficient, such as 14.92%, reflects a relatively equitable distribution of costs among individuals. Several factors could contribute to this outcome. For instance, if respondents live in similar types of housing with comparable heating requirements, their expenses might naturally converge. Additionally, regulated or subsidized heating costs in the region could reduce disparities. Furthermore, widespread use of similar heating systems among respondents might also result in more consistent heating expenses.

The low Gini coefficients for household electricity expenses (12.82%), natural gas expenditures (8.83%), and heating costs (14.92%) indicate relatively low levels of inequality in these specific areas of household spending. When compared to broader economic measures such as the country’s income inequality or Gini coefficients for other types of expenditures, these values highlight a notable uniformity in energy-related costs among households. Economically, these findings suggest that energy costs are fairly evenly distributed, reflecting affordability and accessibility across different income groups. For electricity, a Gini coefficient of 12.82% indicates that electricity expenses are relatively consistent cross households. This could imply that electricity pricing policies or subsidies ensure affordability for most, thereby minimizing disparities in consumption and expenditure. Similarly, the even lower Gini coefficient of 8.83% for natural gas expenditures points to an even more uniformly affordability and accessibility, suggesting that natural gas is priced or regulated in a way that enables households of varying incomes to maintain similar consumption patterns. Heating costs, with a slightly higher Gini coefficient of 14.92%, still reflect a relatively low level of inequality. This may be influenced by similar housing types or heating needs among respondents, as well as regulatory frameworks that standardize heating costs. In comparison to the country’s income inequality, these low Gini coefficients underline that energy expenditures are much less unequal, highlighting the success of pricing mechanisms, subsidies, or other measures that promote equitable energy access.

**Fig. 4:**
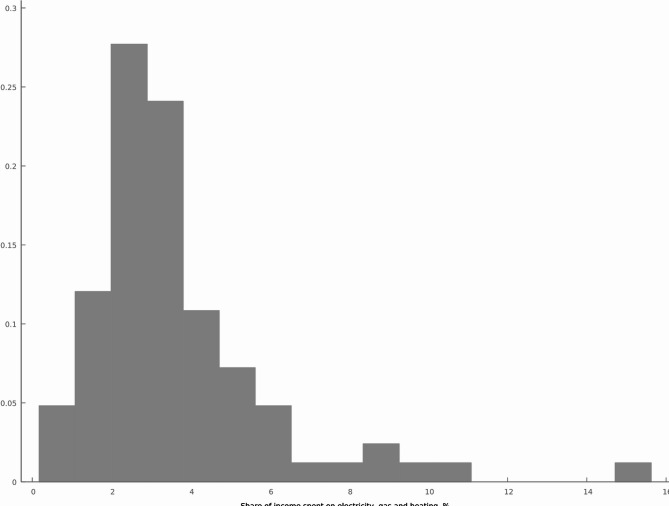
Share of household income allocated to expenses for electricity, gas, and heating: income proportion and spending patterns.

Additionally, the survey assessed the proportion of respondents’ income allocated to taxes payable for electricity, gas, and heating. Figure 4, analyzing the relative frequency of the provided answers, reveals the fluctuations are not very pronounced, with the maximum portion of taxable payments within respondents’ income not exceeding 27%. This observation aligns with the reported distribution of monthly net household income per member, where the largest share of respondents falls within the €1,201–€1,800 (23.4%) and €1,801 or more (26.4%) income brackets, followed by smaller proportions in lower income groups: €801–€1,200 (18.9%), €501–€800 (15.8%), and up to €500 (12.4%). A small percentage of respondents (3.1%) chose not to disclose their income. The relatively equitable income distribution within the surveyed population helps explain why respondents allocate no more than 27% of their income to utilities. With a significant proportion of households earning moderate to higher incomes, utility costs as a percentage of income remain relatively stable Moreover, households may adjust their consumption to match their income and utility costs, such as reducing heating during colder months or adopting energy-saving practices, contributing further to this stability. Together, these factors highlight why the portion of income spent on utilities remains within a narrow range and does not exceed 27% of respondents’ income.

Homogeneity in consumption patterns also contributes to these low Gini coefficients. If households have similar energy needs and usage habits, their expenditures on electricity, natural gas, and heating are likely to be comparable. This could be due to similar household sizes, living conditions, and energy consumption behaviors across the population. Despite the low Gini coefficients indicating low inequality in specific energy expenditures, energy inequality can still exist in other dimensions. Access to different energy sources can vary, with some households lacking access to renewable energy options or more efficient technologies, which provide long-term savings and environmental benefits. Moreover, the concept of energy burden, which refers to the proportion of household income spent on energy, can reveal hidden inequalities. Low-income households might experience higher energy burdens even if their absolute expenditures are similar to those of higher-income households, leading to financial stress and energy inequality. Quality of energy services is another aspect where inequality might be present. Households in underdeveloped areas might face frequent outages or poor-quality energy services, contributing to inequality in energy access and quality. Geographical disparities also play a role, with rural areas potentially facing higher energy costs or limited access to certain energy types compared to urban areas.

In overall, the low Gini coefficients for electricity, natural gas, and heating costs suggest a generally equitable distribution of these energy expenditures among households. The Gini coefficients reflect how evenly the reported expenditures are distributed among those who use these energy sources and have provided their expenditure data. These low values indicate that within this group, energy costs are fairly uniform, implying that most households spend similar amounts on these utilities. This uniformity could be due to effective energy policies, subsidies, and widespread access to affordable energy sources, which help to stabilize prices and make them accessible to a broad spectrum of households. However, focusing solely on Gini coefficients can obscure other forms of energy inequality. For instance, not all households may have equal access to different types of energy. Some households might lack access to natural gas or more efficient energy sources, which can lead to higher relative energy costs or limited energy options, contributing to energy inequality.

Energy burden, which refers to the proportion of household income spent on energy, can also reveal hidden inequalities. Even if absolute expenditures are similar, low-income households might spend a higher percentage of their income on energy, leading to financial stress and higher energy burdens. This indicates energy inequality despite low expenditure inequality. The quality and reliability of energy services can vary significantly. Households in underdeveloped or rural areas might experience frequent power outages, lower-quality energy services, or higher costs for the same services compared to urban areas. These geographical disparities contribute to energy inequality that is not captured by the Gini coefficients. Moreover, not all households have the means to invest in energy-efficient appliances or home improvements. Consequently, some households may spend more on energy due to inefficient usage, leading to higher overall costs and inequality in energy consumption and expenditures. Access to affordable and reliable energy has long-term impacts on health, education, and economic opportunities. Households struggling with high energy costs may face additional challenges, such as inadequate heating or cooling, which can affect their overall quality of life and long-term socioeconomic status.

The low Gini coefficients for each energy source suggest that the distribution of energy costs in Lithuania is relatively equitable, as evidenced by the analysis of household expenditures on electricity, natural gas, and heating. This implies that the financial burdens associated with energy expenses are consistent across the majority of households, indicating that they are widely accessible and affordable. Nevertheless, the uniformity in expenditure distribution suggests that energy costs are equitable; however, it is crucial to acknowledge that energy inequality may continue to exist in other domains, such as access to renewable energy technologies, energy quality, or geographical disparities. Consequently, the results suggest that the energy expenditure landscape is generally equitable. However, policymakers must persist in addressing more profound forms of energy inequality to guarantee that all households have access to sustainable, affordable, and reliable energy services.

## Balancing green ambitions with economic realities: public opinion on renewable energy

This research investigates public attitudes toward renewable energy by conducting a representative survey that concentrates on the perceived benefits, barriers, and willingness to adopt these technologies. This method allows for a comprehensive evaluation of the extent to which climate objectives are in accordance with the current levels of energy inequality and public attitudes. Furthermore, a recent survey was conducted to determine the public’s perspective on a variety of topics, including sustainable living practices, energy efficiency, and renewable energy. The results offer a preliminary assessment of the perspectives of individuals regarding the economic trade-offs associated with environmental responsibility, which is beneficial in assessing the feasibility of establishing a climate-neutral society and the additional measures required to guarantee a sustainable future for future generations.

When asked whether they would agree to pay more for electricity produced from renewable energy sources, the majority of respondents (75.9%) indicated they would not be willing to pay extra. This suggests that while there is general support for renewable energy in principle, many individuals are hesitant to bear the immediate financial burden. However, nearly one-quarter (23.9%) of respondents expressed a willingness to pay more, though their willingness was generally modest. Specifically, 19.6% would agree to pay up to 5% more, 3.8% would agree to a 5–10% increase, 0.3% would agree to a 11–25% increase, and only 0.2% would be willing to pay more than 25% extra. The small fraction who did not specify an answer (0.2%) suggests a slight uncertainty or indecision among the population. Economically, these findings reflect the principle of price elasticity of demand. The low willingness to pay significantly more for renewable energy indicates a relatively elastic demand for electricity, where consumers are sensitive to price increases. The modest willingness to pay slightly more could be interpreted as a recognition of the environmental benefits, but it also underscores the importance of keeping energy costs affordable.

The survey also asked respondents if they would agree to reduce their household energy consumption as a means to combat climate change, for example, by lowering home temperatures or doing laundry less frequently. A significant majority (61.3%) were not willing to make such reductions, highlighting the challenge of encouraging voluntary changes in consumption behavior. Of those willing to reduce consumption, 32.1% would only agree to a slight reduction (up to 5%), while smaller percentages would agree to more substantial cuts—5% would reduce consumption by 5–10%, 0.9% by 11–15%, and only 0.4% by more than 25%. The reluctance to reduce consumption, even slightly, underscores that maintaining comfort and convenience takes precedence over personal contributions to climate change mitigation. This indicates a general resistance to voluntary behavioral changes that disrupt established energy consumption patterns. For policy design, this suggests that solutions requiring individuals to adjust their consumption patterns are likely to face resistance. Therefore, climate mitigation strategies should account for this reluctance by either implementing measures that do not rely heavily on behavioral changes or by introducing incentives that make reductions in energy consumption more attractive and manageable for households. Policies may also need to incorporate public education and awareness campaigns to emphasize the collective impact of individual efforts in combating climate change.

When it comes to purchasing appliances, the survey revealed that an overwhelming majority (84.8%) prefer energy-efficient options. This preference could be driven by the long-term cost savings associated with lower energy bills, in addition to environmental considerations. Meanwhile, 14.8% of respondents did not consider energy efficiency a priority, potentially due to the higher upfront costs of energy-efficient appliances or a lack of awareness of the potential savings. A small percentage (0.4%) did not provide an answer. The use of energy-saving light bulbs is another area where most respondents (90%) are aligned with energy-efficient practices. The near-universal adoption of these bulbs may be due to their proven cost-effectiveness over time, despite their higher initial price. The 9.6% who do not use such bulbs may be influenced by factors such as cost, availability, or lack of information about the benefits. Again, a small percentage (0.4%) did not respond.

Conservation of resources, such as water, heating, and electricity, was reported by a majority of respondents (81.9%), indicating a high level of engagement in resource-saving practices at home. Conversely, 17.7% reported that they do not conserve these resources, while a small portion (0.4%) did not provide an answer. To better understand how conservation behaviors intersect with other attitudes and actions, such as willingness to pay more for cleaner energy or willingness to reduce energy consumption, future analysis could explore the overlap between these factors. For instance, examining whether individuals who actively conserve resources are also more likely to support cleaner energy initiatives could help identify environmentally cautious consumers. Such intersections could provide a clearer picture of how aligned attitudes and behaviors are among respondents.

The survey also revealed that a strong majority (86.4%) of respondents actively sort their waste, demonstrating a commitment to recycling and environmental stewardship. However, 13.2% do not engage in waste sorting, which may point to barriers such as lack of infrastructure, inconvenience, or insufficient motivation. A small fraction (0.4%) did not specify their behavior.

When asked about the potential use of renewable energy sources in their homes for heating, cooling, hot water, and electricity generation, 73.7% of respondents expressed interest. This indicates a general openness to transitioning to renewable energy, likely driven by both environmental concerns and potential long-term cost savings. On the other hand, 25.7% were not interested, possibly due to perceived high upfront costs, lack of access, or skepticism about the benefits. A small percentage (0.6%) did not provide an answer.

Regarding vehicle ownership, a large majority (93.2%) reported that they do not currently drive a new, efficient, and low-emission car. This is likely due to the higher purchase price of such vehicles, despite the potential for savings on fuel and maintenance. However, when asked if they would like to drive such a vehicle, 68.3% said they would, suggesting that cost is a significant barrier to adoption. The 31.3% who would not prefer such a car may be influenced by factors such as brand loyalty, performance concerns, or lack of infrastructure for electric vehicles. A small percentage (0.4%) did not provide an answer.

Finally, the survey explored sustainable living practices when purchasing products. The responses were nearly evenly split, with 48% considering sustainable practices and 51.6% not prioritizing them. This divide suggests that while there is growing awareness of sustainability, convenience, price, and other factors still play a significant role in consumer decision-making. The small portion (0.4%) that did not provide an answer may reflect uncertainty or lack of strong opinions on the matter.

The survey results reveal a complex but revealing picture of public attitudes towards sustainability and the transition to a climate-neutral society. While there is broad support for environmentally friendly practices, there are significant economic and behavioral barriers that could slow progress. A majority of respondents are hesitant to pay more for renewable energy, reflecting the challenge of balancing environmental goals with economic realities. This reluctance highlights the importance of making renewable energy more affordable and accessible to encourage broader adoption. Similarly, while many people support the idea of energy efficiency—evidenced by the widespread use of energy-saving light bulbs and a preference for energy-efficient appliances—there is resistance to making more substantial changes, such as reducing household energy consumption. This indicates that while small, cost-effective changes are acceptable to most, larger sacrifices are less popular, which could hinder efforts to reduce overall energy demand. The strong interest in using renewable energy at home and driving low-emission vehicles suggests a readiness for change, but the high costs associated with these technologies are likely barriers that need to be addressed through incentives, subsidies, and further innovation.

Overall, the results point to a population that is generally supportive of moving towards a climate-neutral society but is constrained by economic factors and a reluctance to compromise on comfort and convenience. To achieve climate neutrality, policymakers will need to focus on making sustainable options both more affordable and appealing, while also educating the public on the long-term benefits of these choices. Bridging the gap between environmental awareness and actionable commitment is essential for the successful transition to a sustainable future.

## Discussion

A fundamental tension between economic realities and environmental principles is revealed by the public’s perspective on energy efficiency and renewable energy systems. Recent surveys indicate that, despite the widespread endorsement of green energy and sustainability as a concept, individuals are hesitant to incur the financial expenses and lifestyle modifications required to effect a substantial transition to a climate-neutral society. This hesitancy is indicative of more profound economic considerations and energy justice concerns, as not all individuals are equally competent or interested in participating in the energy transition^24,37–39^.

One significant discovery is that a substantial proportion of respondents (75.9%) are averse to pay a premium for electricity produced from renewable sources. This exemplifies a fundamental economic concern: the price elasticity of demand. Even when price increases are associated with socially advantageous outcomes, such as the reduction of carbon emissions, individuals are exceedingly susceptible to them^40–42^. The majority of respondents were only prepared to accept modest increases in energy costs, and only 23.9% were open to paying more. This reluctance implies that a significant number of individuals prioritize immediate economic concerns over long-term environmental benefits, particularly given that household energy expenditures already consume a relatively substantial portion of disposable income^43,44^.

It is intriguing that this conduct is in stark contrast to the widespread endorsement of energy-efficient products. Energy-efficient appliances were favored by an overwhelming majority (84.8%), and 90% of respondents reported using energy-saving light bulbs. These decisions are indicative of a cost-benefit analysis in which the initial expenses are mitigated by the long-term savings on energy bills, thereby increasing their appeal to consumers. The preference for actions that generate immediate, personal financial gains, as opposed to abstract, collective benefits, is indicated by the disparity in willingness to invest in energy efficiency versus pay a premium for renewable energy. This is evidenced by the reluctance to reduce energy consumption, as 61.3% of respondents were unwilling to decrease their domestic energy consumption, even if such reductions could aid in the fight against climate change.

Conversely, the distribution of household energy expenditures indicates that energy costs are relatively equitable. The low Gini coefficients for residential electricity expenses (12.82%), natural gas expenditures (8.84%), and heating costs (14.92%) suggest that these expenses are fairly uniformly distributed across households. This implies that energy sources are generally affordable and accessible, which contributes to a relatively equitable distribution of financial burdens associated with these utilities. Effective pricing and accessibility are likely to be the reasons why households, irrespective of their income levels, experience comparable energy costs. Nevertheless, the complexities of energy justice are not negated by this general equity in expenditure. Energy inequality persists in other dimensions, despite the low Gini coefficients, which suggest a generally equitable distribution of costs. For instance, lower-income households may have restricted access to renewable energy sources or more energy-efficient technologies, which can lead to increased relative energy costs and less favorable energy outcomes for these populations. Furthermore, the concept of energy burden, which involves low-income households allocating a greater proportion of their income to energy, underscores concealed disparities that are not entirely represented by Gini coefficients.

The results of the survey indicate that consumers are more likely to respond to incentives that directly affect their finances in an economic sense. The advantage of implementing energy-efficient appliances or light bulbs is evident: reduced energy costs^45^. Nevertheless, the benefits of renewable energy are more dispersed throughout society, resulting in less tangible personal rewards for those who pay a premium for it^46^. This emphasizes the necessity for policymakers to increase the financial accessibility of renewable energy, potentially through subsidies or reimbursements, in order to address this reluctance.

These discoveries are alarming from the standpoint of energy justice. The risk that the benefits of renewable energy will be concentrated among wealthier households who can afford the higher costs is underscored by the hesitancy to pay more for green energy, leaving lower-income populations dependent on less sustainable, cheaper energy sources^47,48^. This would exacerbate preexisting inequalities, resulting in an unequal distribution of the economic and environmental advantages of the energy transition.

Simultaneously, the widespread adoption of energy-efficient technologies such as light bulbs and appliances indicates that individuals are more inclined to make environmentally friendly decisions when the economic incentives are evident. However, energy justice concerns persist, as not all households are able to afford the initial expenses of energy-efficient appliances, despite the substantial long-term savings. It will be essential to address these economic disparities by implementing targeted programs that assist low-income households in the adoption of these technologies in order to achieve a fair energy transition. The disparity in energy consumption also underscores the necessity of incorporating energy justice into policies that are designed to decrease the overall energy demand^24,49,50^. In order to alter behavior, it will be necessary to implement more aggressive policies or incentives, as a significant number of respondents are averse to voluntarily reduce their energy consumption. Nevertheless, it is crucial that these policies are implemented in a manner that does not disproportionately affect those who are already grappling with energy costs.

The survey findings indicate that the general population is in favor of environmental objectives, but they are constrained by economic considerations. Despite a distinct preference for energy-efficient products that provide personal financial savings, there is a reluctance to make larger sacrifices, such as paying a premium for renewable energy or reducing energy consumption, where the benefits are less immediately apparent. This underscores the necessity for policymakers to reconcile environmental objectives with economic realities, particularly by means of financial incentives that render sustainable alternatives more affordable. Furthermore, it is imperative to prevent these policies from exacerbating existing inequalities in order to establish a climate-neutral and equitable society.

## Conclusion

A comprehensive approach integrating energy justice with climate action is essential to achieving European Union’s ambition of climate neutrality by 2050. This study highlights a significant discrepancy between environmental principles and economic realities. While there is broad support for energy efficiency and sustainability, many individuals remain reluctant to incur additional costs for renewable energy and make significant lifestyle changes. This reluctance reflects deeper economic concern where immediate financial impacts often outweigh long-term environmental benefits.

Low Gini coefficients for electricity, natural gas, and heating expenditures in Lithuania suggest a generally equitable distribution of energy costs across households. However, this uniformity masks hidden inequalities, such as higher energy burdens on low-income households, limited access to renewable energy technologies, and geographical disparities in energy quality and availability. These findings underscore the need for more targeted interventions to address these inequalities, particularly for rural and economically vulnerable populations.

The study also reveals shared economic motivations driving energy-related decisions. While high adoption rates of energy-efficient technologies highlight the appeal of tangible financial benefits, the reluctance to pay premiums for renewable energy underscores the economic constraints faced by many households. These findings emphasize the importance of financial incentives, such as subsidies and tax credits, to enhance the accessibility of renewable energy without disproportionately burdening lower-income groups.

Addressing both the economic and social dimensions of energy inequality is crucial for a fair and inclusive energy transition. Policies must prioritize equitable access to energy resources and technologies while ensuring that sustainable options are both affordable and appealing. Lithuania’s context—marked by a mix of urban and rural populations, disparities in energy access, and evolving energy infrastructure—provides valuable insights that are applicable to other EU countries facing similar challenges. To bridge the gap between environmental awareness and actionable commitment, the EU must align environmental objectives with economic realities and implement policies that address hidden inequalities. These efforts will ensure that the transition to climate neutrality is both sustainable and equitable, leaving no community behind and fostering a shared commitment to protecting the planet for future generations.

### Limitations

This research on energy inequality and public attitudes toward renewable energy is subject to several limitations. Although the survey was designed to be representative, the generalizability of the results may be affected by sample bias, including the potential underrepresentation or overrepresentation of certain demographic groups. Moreover, self-reported data on household energy expenditures and attitudes may be influenced by recall inaccuracies, misinterpretation of questions, or respondents’ reluctance to disclose financial information. The analysis does not fully account for the impact of changing economic conditions or fluctuations in energy prices, which may alter both household expenditures and public sentiment over time. Regional disparities in energy costs and access—particularly between urban and rural areas—may also be insufficiently captured. In addition, the study does not incorporate differences in energy service quality or household energy efficiency, both of which are important dimensions of energy justice. The survey may also underrepresent the complexity of energy burdens experienced by lower-income households. Methodologically, while the Gini coefficients for electricity, natural gas, and heating expenditures suggest relatively low inequality, this measure has well-known limitations. The Gini coefficient reflects the distribution of spending but does not account for affordability, energy needs, or access to renewable technologies. Consequently, it may underestimate the true extent of energy inequality—particularly in cases where households with similar expenditure levels differ significantly in income, vulnerability, or housing quality. Furthermore, the Lorenz curves in this study are constructed based on energy expenditure rankings rather than income deciles. Although this is a valid and commonly used method in energy justice research, it may yield lower inequality estimates than income-based approaches and could obscure the disproportionate burden faced by lower-income households. A complementary analysis based on income deciles would enhance the understanding of affordability and is recommended for future research. Overall, these limitations underscore the importance of adopting a multidimensional approach that includes expenditure, income, energy efficiency, service quality, and access to clean technologies to more fully assess and address energy inequality.

## Data Availability

The data that support the findings of this research are available from the corresponding author upon reasonable request.
